# Supplementary material for: Splice-Junction-Based Mapping of Alternative Isoforms in the Human Proteome
Source: Cell Rep. Author manuscript; Available in PMC 2020 Jan 15. (PMC6961840; doi:10.1016/j.celrep.2019.11.026)
Supplement: 2 [file NIHMS1546469-supplement-2.zip › DF1/PXD010154/Spleen/Spleen_12_MAPKAPK5_QVIEEQTTSHESTGGAK.pdf]

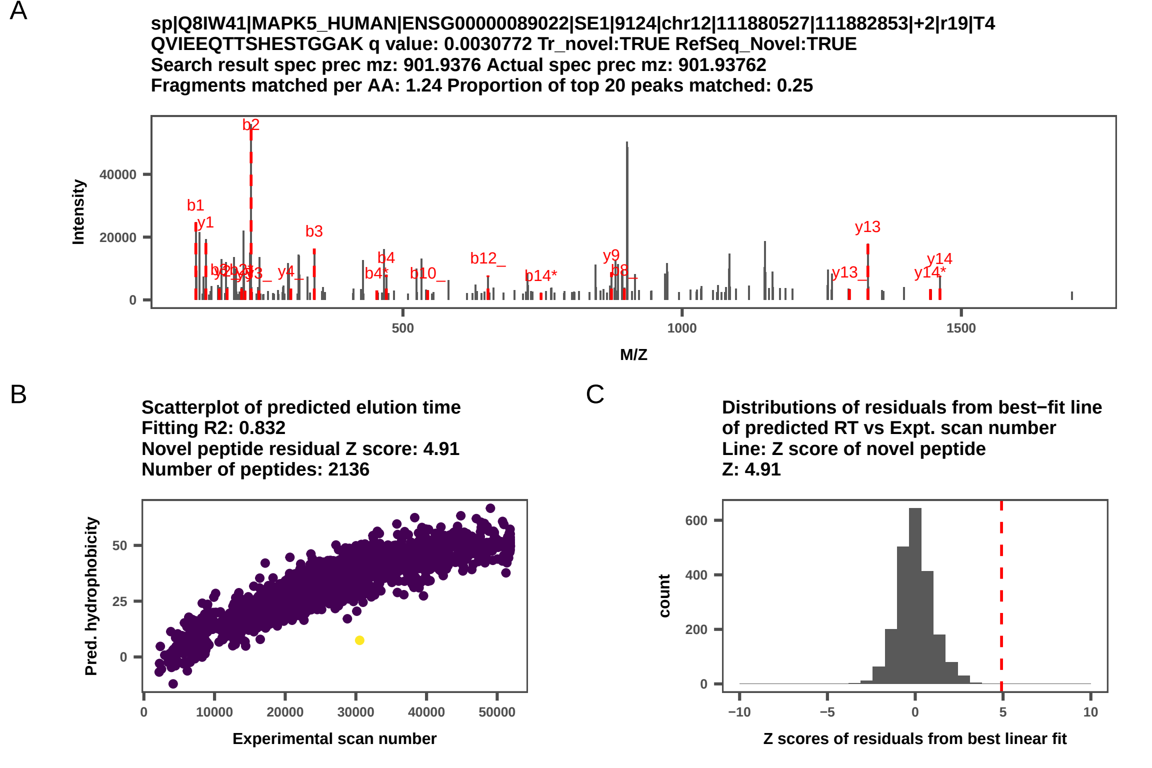
A
sp|Q8IW41|MAPK5_HUMAN|ENSG00000089022|SE1|9124|chr12|111880527|111882853|+2|r19|T4
QVIEEQTTSHESTGGAK q value: 0.0030772 Tr_novel:TRUE RefSeq_Novel:TRUE
Search result spec prec mz: 901.9376 Actual spec prec mz: 901.93762
Fragments matched per AA: 1.24 Proportion of top 20 peaks matched: 0.25
Intensity
0
20000
40000
b2
b1
y1
b3
b4
b4*
b10_
b12_
b14*
y9
b8_
y13
y13_
y14
y14*
y4_
500
1000
1500
M/Z
B
Scatterplot of predicted elution time
Fitting R2: 0.832
Novel peptide residual Z score: 4.91
Number of peptides: 2136
Pred. hydrophobicity
0
25
50
0
10000
20000
30000
40000
50000
Experimental scan number
C
Distributions of residuals from best−fit line
of predicted RT vs Expt. scan number
Line: Z score of novel peptide
Z: 4.91
count
0
200
400
600
−10
−5
0
5
10
Z scores of residuals from best linear fit
